# CRISPR/Cas9-mediated mutagenesis of the *dihydroflavonol-4-reductase-B (DFR-B)* locus in the Japanese morning glory *Ipomoea (Pharbitis) nil*

**DOI:** 10.1038/s41598-017-10715-1

**Published:** 2017-08-30

**Authors:** Kenta Watanabe, Anna Kobayashi, Masaki Endo, Kimiyo Sage-Ono, Seiichi Toki, Michiyuki Ono

**Affiliations:** 1 Graduate School of Life and Environmental Sciences, University of Tsukuba, 1-1-1 Tennodai, Tsukuba, Ibaraki, 305–8572 Japan; 2 College of Biological Sciences, School of Life and Environmental Sciences, University of Tsukuba, 1-1-1 Tennodai, Tsukuba, Ibaraki, 305–8572 Japan; 3 Plant Genome Engineering Research Unit, Institute of Agrobiological Sciences, National Agriculture and Food Research Organization (NARO), 2-1-2 Kannondai, Tsukuba, Ibaraki, 305–8602 Japan; 4 Gene Research Center, Tsukuba Plant Innovation Research Center (T-PIRC), University of Tsukuba, 1-1-1 Tennodai, Tsukuba, Ibaraki, 305–8572 Japan; 5 Graduate School of Nanobioscience, Yokohama City University, 22–2 Seto, Kanazawa-ku, Yokohama, Kanagawa 236–0027 Japan; 6 Kihara Institute for Biological Research, Yokohama City University, 641–12 Maioka-cho, Yokohama, Kanagawa 244–0813 Japan

## Abstract

CRISPR/Cas9 technology is a versatile tool for targeted mutagenesis in many organisms, including plants. However, this technique has not been applied to the Japanese morning glory (*Ipomoea [Pharbitis] nil)*, a traditional garden plant chosen for the National BioResource Project in Japan. We selected *dihydroflavonol-4-reductase-B* (*DFR-B*) of *I. nil*, encoding an anthocyanin biosynthesis enzyme, as the target gene, and changes in the stem colour were observed during the early stages of plant tissue culture by *Rhizobium [Agrobacterium]*-mediated transformation. Twenty-four of the 32 (75%) transgenic plants bore anthocyanin-less white flowers with bi-allelic mutations at the Cas9 cleavage site in *DFR-B*, exhibiting a single base insertion or deletions of more than two bases. Thus, these results demonstrate that CRISPR/Cas9 technology enables the exploration of gene functions in this model horticultural plant. To our knowledge, this report is the first concerning flower colour changes in higher plants using CRISPR/Cas9 technology.

## Introduction

Methods for targeted mutagenesis have rapidly been developed, and their applications to exotic plants are gradually advancing^1, 2^. Several systems have been developed to induce DNA double-strand breaks (DSBs) at specific genome sites. Such breaks provide increased opportunities to induce site-directed mutations through DNA repair systems, such as non-homologous end joining (NHEJ)^3^. Among engineered nuclease systems for DSBs, the clustered regularly interspaced short palindromic repeat (CRISPR)-associated endonuclease 9 (Cas9) system can be applied more readily than other systems because of its simple experimental design and high specificity^4^. The CRISPR/Cas9 system has been successfully utilized for targeted mutagenesis in a variety of organisms, including plants, and will increase reverse genetic studies, particularly for plants with good transformation systems and high-quality genome sequences.

The Japanese morning glory, *Ipomoea nil* or *Pharbitis nil*, is one of two traditional horticultural model plants selected for the National BioResource Project in Japan by the Agency for Medical Research and Development (AMED)^5^. A high-quality genome sequence of *I. nil* has recently been reported, with a scaffold N50 of 2.88 Mb, covering 98% of the 750 Mb genome^6^. Because we previously established and improved its transformation methods^7, 8^, this plant is also a suitable target for the CRISPR/Cas9 system. *I. nil* was imported from China to Japan in the eighth century as a medicinal plant but quickly became a traditional garden plant in Japan. Currently, Asagao (*I. nil*, in Japanese) is a representative of the summer garden flower, and most Japanese elementary students grow and observe this species as their first experimental plant^6^. Studies on Asagao are welcomed not only by scientists but also by Japanese citizens. Currently, public concern over new plant breeding technology (NBT) is a social issue in Japan. Therefore, studies on the *I. nil* genome will provide an excellent opportunity to facilitate science literacy for the public understanding of NBT.

During the Edo period (approximately 200 years ago), explosive spontaneous transposon mutagenesis occurred in *I. nil* cultivars. The resulting mutated *I. nil* plants, with various colours and shapes, primarily in flowers, were collected, and the cultivation and competition of these mutants repeatedly occurred^6^. Since the 1910s, extensive genetic studies have been conducted on these mutants, and the resulting chromosome map was the most advanced in the plant kingdom at that time^9, 10^. Currently, *I. nil* is the model horticultural plant in many respects, not only for basic studies of floricultural traits, such as flower colour^11, 12^, flower pigmentation patterns with transposon mutagenesis^13, 14^ and flower shape^15, 16^, but also for studies on physiological phenomena, such as phytohormones^17^, gravitropism^18^, petal senescence^19^, and others. As *I. nil* is a short-day plant and the seedling of the ‘Violet’ cultivar is an absolute short-day plant^20^, extensive studies have been conducted using these species^21–25^. Moreover, from the perspective of gene duplication and evolution, the *dihydroflavonol-4-reductase* (*DFR*) gene family has been well characterized as reflecting escape from adaptive conflict (EAC)^26^. Thus, the history of *I. nil* makes this species an outstanding model plant.

Since initiating the National BioResource Project (NBRP) in Japan^5^, libraries of expression sequence tags (ESTs) and bacterial artificial chromosomes (BACs) have been constructed^6^, and genetic and molecular markers have been prepared^27^. These resources make *I. nil* an ideal model plant for basic and horticultural studies in physiology and molecular biology. The establishment of targeted mutagenesis in this plant will significantly improve the research environment and will highlight the research sources in the NBRP “Morning glory”.

To confirm the applicability of the CRISPR/Cas9 system to *I. nil*, we selected *dihydroflavonol-4-reductase-B* (*DFR-B*), the gene encoding an enzyme in the anthocyanin biosynthesis pathway^28^, and CRISPR/Cas9 system-mediated changes in the stem colour were observed during the early stages of plant tissue culture via *Rhizobium*-mediated transformation. Moreover, in morning glories, including *I. nil*, *DFR* is present as a small, tandemly arrayed three-gene family (*DFR-A*, *DFR-B* and *DFR-C*), although most Solanaceae species, the relatives of Convolvulaceae, including *Ipomoea*, have a single copy of the *DFR* gene^29^. In the common morning glory, *Ipomoea purpurea*, all three genes are expressed, but *DFR-B* is the main gene and is interpreted as EAC. Moreover, in *I. nil*, these three genes are structurally normal, but *I. nil DFR-B* (*InDFR-B*) is the genetically dominant gene responsible for pigmentation in the stems and flowers, as several spontaneous mutants of *InDFR-B* have shown the null phenotype^30^. Thus, it remains unknown whether the targeted mutagenesis of *InDFR-B* located between *InDFR-A* and *InDFR-C* causes the null phenotype. Additionally, to confirm the accuracy of the CRISPR/Cas9 system, potential off-target modifications on the two orthologous genes, namely, *DFR-A* and *DFR-C*, were examined. These observations reconfirm the importance of the protospacer adjacent motif (PAM) in targeted mutagenesis using the CRISPR/Cas9 system in *I. nil*. The Cas9 protein is an endonuclease functioning with single guide RNA (sgRNA). The Cas9, sgRNA complex scans double-stranded DNA to detect DNA sequences complementary to the 20-nucleotide (nt) target sequence in the sgRNA and the NGG motif, referred to as the PAM, located immediately after the target sequence. The PAM is essential for the binding of CRISPR/Cas9 to the DNA target^31, 32^.

The first spontaneous white flower mutant in *I. nil* was painted in 1631 in Japan, approximately 850 years after the initial import of the blue wild-type flower plants from China. Using the CRISPR/Cas9 system, within a year, we generated several white flower mutants at the same locus but affecting different alleles, indicating the strength of this system and its future prospects. To our knowledge, this study is the first to establish changing flower colour in higher plants using the CRISPR/Cas9 system.

## Results

### Selection of the target gene and the sgRNA for CRISPR/Cas9

**Figure 1:**
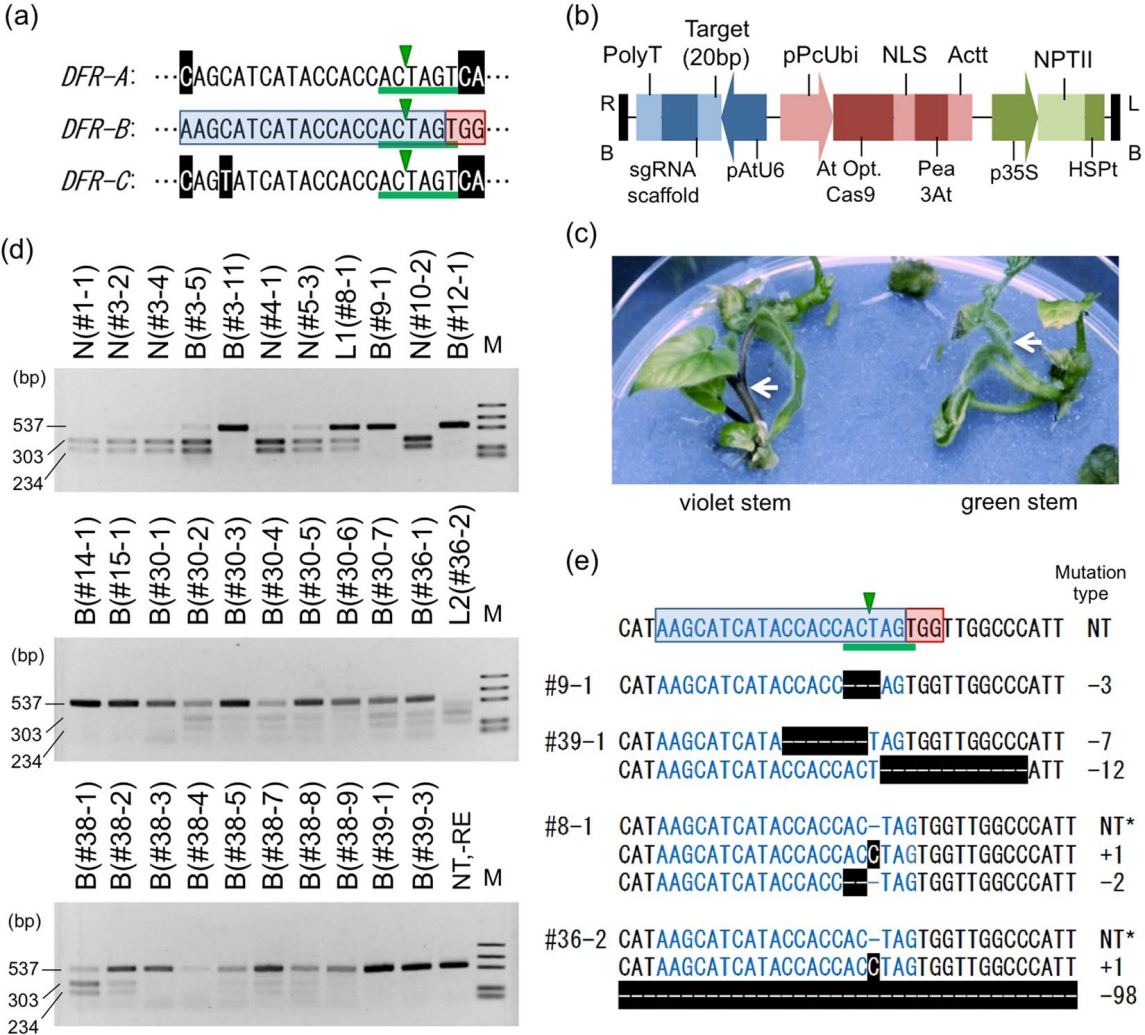
CRISPR/Cas9-mediated targeted mutagenesis in *InDFR-B*. (**a**) Schematic representation of *InDFR-A, -B*, and *-C* target sequences. In *InDFR-A* and *-C*, the white letters in black highlight indicate mismatches with *InDFR-B*. In *DFR-B*, the 20-bp target-specific sequence is shown in blue highlight, and the PAM sequence (TGG) is shown in red highlight. *Spe*I restriction enzyme sites (ACTAGT) are underlined with green. The green triangles indicate the expected cleavage site of the CRISPR/Cas9 system. (**b**) T-DNA region of the all-in-one vector, pZD_AtU6gRNA_FFCas9 _NPTΙΙ. (**c**) Kanamycin-resistant regenerated shoots of plants transformed with the CRISPR/Cas9 system. Without targeted mutations, stems were coloured violet (left), whereas bi-allelically mutated stems remained green (right). (**d**) CAPS analysis of the target region in the *InDFR-B* locus. Total DNA was extracted from the leaves of transgenic plants and amplified by PCR. The PCR products were digested with the *Spe*I restriction enzyme, except -RE. M: marker (1,000, 700, 500, 200 and 100 bp); NT-RE: PCR product of a non-transgenic (NT) plant without restriction enzyme digestion. Numerals after # indicate independent T1 plants. N, B, L1, and L2 represent the phenotype of each plant. N: Violet stem and violet flower (same as NT); B: Green stem and white flower; L2: Green stem and violet flower; L1: Violet stem and pale-violet flower. (**e**) Sequences of targeted mutations in the *InDFR-B* locus. The NT type sequence is shown at the top and is designated (**a**). Deleted nucleotides are shown in dashes with black highlight (-). The inserted cytidine residue is shown with black highlight. NT sequences were detected using CAPS analysis (*) in chimaeric plants (#8-1 and 36-2).

To examine whether the CRISPR/Cas9 system could be applied to *I. nil*, we selected the *InDFR-B* gene [accession number: AB006793]^29^ as the target of Cas9 endonuclease. Because null mutations in *InDFR-B* lead to anthocyanin-less stems, leaves and flowers, we visually distinguished the bi-allelic mutants during transformation. For the sgRNA sequence, we selected 20 bp in the fourth exon of the *InDFR-B* gene encoding a catalytic site of the DFR enzyme (Fig. 1a)^33^. The sgRNA sequence of *InDFR-B* shows high homology to those of *InDFR-A* and *InDFR-C*, with 19/20 and 18/20 matches in the nucleotide sequences, respectively, but only *InDFR-B* has the PAM next to the sgRNA sequence (Fig. 1a). Potential off-target sites of *InDFR-B* were searched with GGGenome (http://gggenome.dbcls.jp/) allowing no mismatch in seed sequence which is the most critical determinant of target specificity^54, 55^ and 3 bp mismatches in 20 bp sequence. Additional nine sites were derived as potential off-target sites (Supplemental Table S1).

### Construct for the CRISPR/Cas9 system and transformation

An *Arabidopsis* codon-optimized *Streptococcus pyogenes* Cas9 expression cassette^34^, an sgRNA expression cassette, and a selective marker (*neomycin phosphotransferase* II: *NPT*II) were combined into a single plant binary vector to form an all-in-one vector for plant transformation (Fig. 1b). *Cas9* was driven by the constitutive *Ubiquitin4-2* promoter from *Petroselinum crispum* (*pPcUbi*)^34, 35^. The *AtU6* promoter was used to express the sgRNA^36, 37^. The construct was introduced to secondary embryos to make stable transgenic *I. nil* using the *Rhizobium* (*Agrobacterium)* method^7, 8^. A total of 32 transgenic plants (T1) showing kanamycin resistance with the T-DNA insertion in the genome were obtained.

### Identification of the mutants by appearance, CAPS analysis and DNA sequencing

Initially, visual screening based on the appearance of pigmentation with anthocyanin was performed. Non-transgenic cv. Violet plants exhibited violet-coloured stems; however, more than one-third of the regenerated plants showed anthocyanin-less stems, typical of the phenotype of null mutations in *InDFR-B* (Fig. 1c). Notably, we never observed a shift in colouring, namely, violet to green/green to violet within a single plantlet and its stems during growth.

Subsequently, we conducted a cleaved amplified polymorphic sequence (CAPS) analysis to detect mutations in the target region. We designed the expected cleavage site of CRISPR/Cas9 overlapping with the recognition sequence of the restriction enzyme *Spe*I (Fig. 1a). If the CRISPR/Cas9 cleaved target sequence and NHEJ occurred, then this restriction site would collapse, and the PCR-amplified DNA fragment would not be digested using *Spe*I. To detect mutations, total DNA was extracted from the transgenic leaves and PCR-amplified, and the resulting PCR products were subjected to *Spe*I digestion and analysed via agarose gel electrophoresis. The non-mutated PCR fragment of *InDFR-B* is 537 bp in length, and *Spe*I digestion produced 303- and 234-bp fragments; however, the mutated PCR fragments were not digested (Fig. 1d). Ten kanamycin-resistant transgenic plants showed *Spe*I-resistant single DNA bands, indicating that these plants are candidates of the bi-allelic mutants. Seventeen plants had both undigested and digested bands (Fig. 1d). Among these mutants, the #36-2 plants showed a shorter DNA fragment approximately 400 bp in length prior to *Spe*I digestion, suggesting a larger deletion in the locus (Fig. 1d). We also analysed the two orthologous loci, namely, *InDFR-A* and *InDFR-C*, using CAPS analysis and detected no mutations (Supplemental Fig. S1).

Moreover, the DNA sequences of the mutated sites in *InDFR-B* were determined. The error-free PCR-amplified fragments of the mono-allelic mutants, #8-1 and 36-2, and the bi-allelic mutants, #9-1 and 39-1, were cloned and subjected to DNA sequencing. All plants showed mutations in the predicted cleavage site (Fig. 1e). The detected insertion was one bp, whereas the deletions ranged from two to 98 bp. Interestingly, the L2 chimaeric plant #8-1 showed three patterns of DNA sequences in *InDFR-B* (two different mutations and one non-mutation), despite the diploid genome. The L1 chimaeric plant #36-2 had the longest deletion (98 bp), as predicted in the CAPS analysis (Fig. 1d,e and Supplemental Fig. S2). Moreover, we analysed all the nine off-target candidates sites having a PAM site listed in Supplemental Table S1 with DNA sequencing in the biallelic mutants #9-1 and 39-1, and found there was no mutation in the candidate sites (Supplemental Fig. S6).

### Phenotype and genotype of the targeted mutagenesis

**Figure 2:**
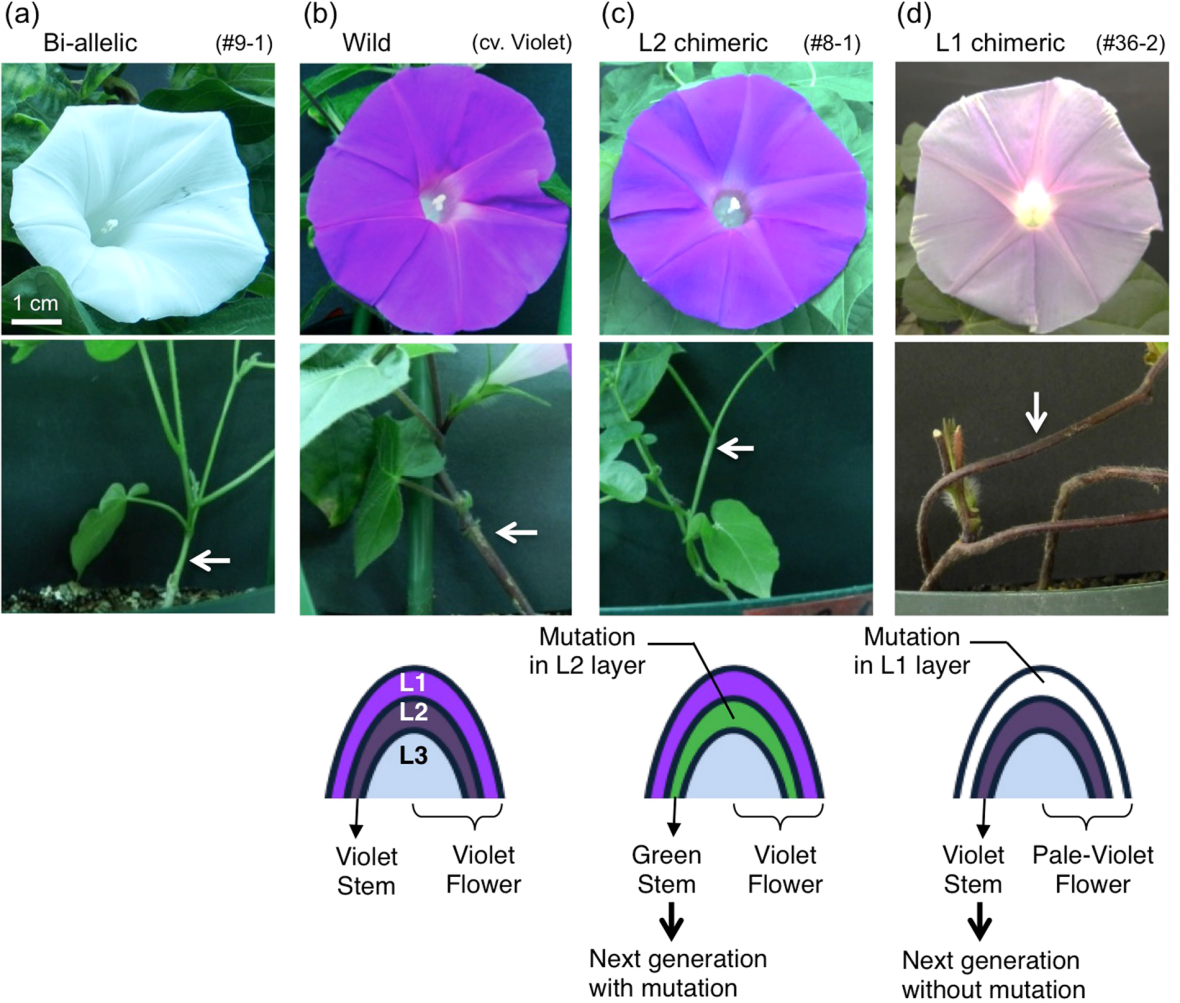
Flowers of CRISPR/Cas9-mediated *dfr-b* mutants. The appearances of flowers (top) and stems (middle) and a schematic drawing of the meristem layers and their functions (bottom). L1: epidermal layer; L2: sub-epidermal layer; L3: internal tissues. (**a**) A flower and stem of #9-1, a bi-allelic-mutant plant. (**b**) A flower and stem of *I. nil* cv. Violet, an NT plant. (**c**) A flower and stem of #8-1, an L2 periclinal chimaera plant and a representation of the meristem layers of the L2 chimaera showing bi-allelic mutation only in the L1 layer. (**d**) The flower and stem of #36-2, an L1 periclinal chimaera plant and a representation of the meristem layers of the L1 chimaera showing bi-allelic mutation only in the L2 layer.

Bi-allelic mutants lost anthocyanin, resulting in green stems and white flowers (Fig. 2a). Non-mutated plants showed violet stems and flowers, similar to non-transgenic plants (NT) (Fig. 2b). Most of the mono-allelic mutants also showed violet stems and flowers, similar to non-mutated plants. Notably, plants #8-1 and #36-2, considered mono-allelic mutants based on CAPS analysis, showed green stems with violet flowers and violet stems with pale flowers, respectively (Fig. 2c,d). These two plants were considered periclinal chimaeras, as these phenotypes were the same as periclinal chimaeras resulting from transposon mutagenesis^30^. In *I. nil*, the L1 layer is responsible for most of the flower colour, whereas the L2 layer determines the stem colour and the genotype of gamete cells^30^. Therefore, plant #8-1 was considered an L2 chimaeric plant with a targeted mutation in *DFR-B* in the L2 layer, and plant #36-2 was considered an L1 chimaeric plant with a targeted mutation in *DFR-B* in the L1 layer. Because the L2 layer slightly contributed to the petal colour, the flowers of plant #36-2 were pale violet, as previously reported^30^. Then we checked the indel mutation and T-DNA existence at the root, stem, leaf and petal of #36-2. The root tissue does not contain L1 and L2 layers, because L3 forms the central tissues including the pith and roots^56^. The root of #36-2 had no mutation and no T-DNA insertion as expected (Supplemental Fig. S5). Therefore, we concluded that plant #36-2 was an L1 periclinal chimaera.

**Figure 3:**
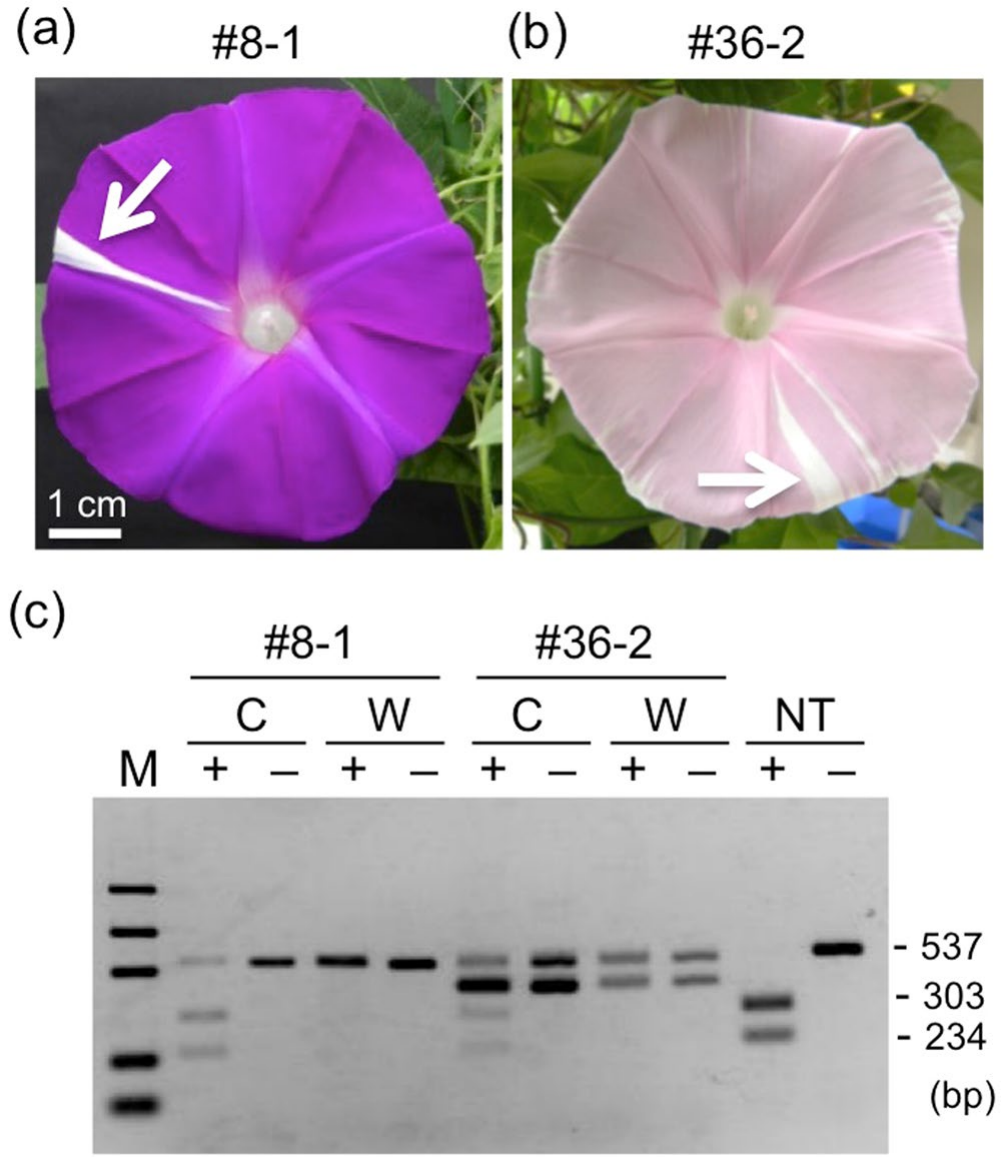
Phenotypes and genotypes of sectorial chimaeric flowers. (**a**) A sectorial flower of the L2 chimaeric plant #8-1. (**b**) A sectorial flower of the L1 chimaeric plant #36-2. (**c**) CAPS analysis of the *InDFR-B* loci in sectorial chimaeric flowers shown in (**a**) and (**b**). Total DNA was extracted from the sectorial white tissues of the petals indicated by arrows (W) and other coloured tissues of the petal (**C**), which were subsequently used for PCR amplification. The PCR products were then digested using the *Spe*I restriction enzyme (+). -: without *Spe*I restriction enzyme digestion; M: marker (1,000, 700, 500, 200 and 100 bp); NT: Total DNA of an NT plant.

We observed few somaclonal changes in anthocyanin pigmentation during cultivation. Periclinal chimaeric plants #8-1 and #36-2, considered L2 and L1 chimaeras, respectively, bore sectorial flowers only once during each life time, baring more than 30 flowers in total (Fig. 3a,b). Sectors of chimaeric plants were extensively studied using transposon mutants in the *DFR-B* locus in *I. nil* ^30^, and two potential causes were considered: somaclonal mutations and invasion of cells from an adjacent layer. We examined the total DNA extracted from sector cells using CAPS analysis and confirmed that these white sectors consisted of bi-allelically mutated cells (Fig. 3c).

### Inheritance of CRISPR/Cas9 system-induced mutations in subsequent generations

We observed the progeny (T2) of the transformed plants, and all results were essentially as expected. For example, the progeny of plant #9-1, bi-allelic mutants, showed green stems (24 plants), indicating that plant #9-1 (T1) was a perfect bi-allelic-targeted mutant of *InDFR-B*. Among the offspring of #9-1, targeted mutants without T-DNA insertions were observed, suggesting that the targeted mutations at *InDFR-B* and T-DNA were not co-inherited in #9-1. These plants are targeted mutants and are considered transgenic plants based on process-based definitions and non-transgenic plants based on product-based definitions^38, 39^.

We also observed the progeny (T2) of the L2 chimaeric mutant #8-1 and the L1 chimaeric mutant #36-2. The progeny of plant #8-1 showed green stems (16 plants), and the progeny of plant #36-2 showed violet stems (50 plants). We examined the T-DNA retention of the 24 progenies of the L1 chimaeric mutant #36-2 using PCR amplification to detect a fragment of the *NPTΙΙ* gene. None of Supplemental Fig. S3 the T2 plants of #36-2 progeny had T-DNA insertions in their genomic DNA (). Because gamete cells originated from L2 cells^30^, these results reconfirmed that plant #8-1 was an L2 chimaeric plant and that plant #36-2 was an L1 chimaeric plant.

## Discussion

These data provide the first demonstration that the CRISPR/Cas9 system can generate targeted mutagenesis in *I. nil*. Approximately one-third of the stable transgenic plants were bi-allelic mutants at the *DFR-B* locus in one generation. This high efficiency indicates that the CRISPR/Cas9 system is a highly applicable technique for next-generation breeding in *I. nil* and other horticultural plants.

We successfully visually selected targeted mutagenesis in the early stage during transformation, as the loss of function of the target gene *DFR-B* prevents the synthesis of stem pigmentation with anthocyanin. In the present study, the gene disruption was detected only at the *InDFR-B* locus, whereas no mutations were observed in the two orthologous loci, namely *InDFR-A* and *InDFR-C*, despite their high homology. These data confirmed the validity of target specificity and the indispensability of PAM for the CRISPR/Cas9 system, avoiding potential off-target mutagenesis. In a recent study, the bi-allelic mutation rate was 31% (10/32), and the total mutation rate was 84% (27/32). The mutation rate of this study was relatively high among higher plants^2, 40^. Moreover, a low tendency of obtaining 6% (2/32) chimaeric plants was observed. Because the transformation and regeneration system in *I. nil* uses secondary embryogenesis from secondary embryos induced on immature embryos, we consider that the consequent plant transformant primarily originated from a single cell of the secondary embryo^7^ except chimaeric plant #8-1 and #36-2. This unique character of the transformation and regeneration system might affect the low tendency of obtaining chimaeric plants. Because growth rate of secondary embryo had an individual difference slightly, some secondary embryos rarely reached to the regeneration step and could be multi-cell embryo rarely, so the chimaeric mutants were obtained at a low tendency. Despite the high efficiency of obtaining bi-allelic mutations, the overall tendency of the results, 1 bp insertions or short deletions of less than 100 bp, was similar to those in other plants, for example, *Arabidopsis* ^41^ and rice^42^. Furthermore, the efficiency of editing depends on sgRNA^2, 42^ and the optimization of sgRNA design to maximize activity is advancing^43^. By selecting a target sequence with optimization program and software^44, 45^, the editing efficiency will increase more in *I. nil*.

In *I. nil*, *DFRs* are present as a small three-gene family (*DFR-A*, *DFR-B* and *DFR-C*). This gene duplication and evolution is the best example of the escape from adaptive conflict (EAC)^26^. In the present study, we successfully inactivated *DFR-B* without modification in *DFR-A* and *DFR-C* genes, although these three genes are tandemly arrayed within a 17-kb DNA region^29^. We reconfirmed that *DFR-B* is the main gene for pigmentation among these three duplicated genes and the EAC in this locus. Using a transposable insertion mutant, a-3′ line of the *DFR-B* locus and its somaclonal revertants, extensive genetic experiments were conducted in 1930s and 1990s^9, 10, 14, 30^. We revisited the same locus in the opposite direction, generating null mutants. This direction, the reverse genetic approach to these legacy phenomena, represents one of the important purposes of targeted mutagenesis.

Plants #8-1 and #36-2, initially classified as mono-allelic mutants as determined by CAPS analysis, showed colour combinations of stems and flowers that differed from those of the other mono-allelic mutants (Fig. 2c,d). Therefore, we considered these plants to be periclinal chimaeric mutants. The shoots of many angiosperms, including *I. nil*, consist of three cell layers: the epidermal cell layer, L1; the sub-epidermal cell layer, L2; and the internal tissues, L3, in the shoot meristems (Fig. 2, below)^46, 47^. In wild-type *I. nil*, flower pigmentation occurs mostly in L1 and slightly in L2, whereas stem pigmentation is restricted to L2^30^. We concluded that these plants were periclinal chimaeric mutants, initially using the results of the comparison of the phenotype with the literature and later using CAPS analysis (Fig. 1d and Supplemental Fig. S5), sequence determination (Fig. 1e and Supplemental Fig. S2), and observation of inheritance in the T2 generation (Supplemental Fig. S3). These results demonstrated plant #8-1 was a periclinal chimaera that had bi-allelic mutation in L2; in contrast, plant #36-2 was also a periclinal chimaera with a bi-allelic mutation in L1 (Fig. 2c,d). Periclinal chimaeric mutations of *DFR-B* in *I. nil* were extensively studied by Imai^9, 10^ with genetic analyses and reanalysed by Inagaki *et al*. in molecular and biochemical studies^30^. Our plants #8-1 and #36-2 exactly followed their reports, particularly regarding the pale flower colour of the L1 chimaera (Fig. 2d). Moreover, plants #8-1 and #36-2 bore sectorial flowers (Fig. 3). Because these sectorial flowers were rare events and difficult to analyse, we could not decide whether these sectors resulted from somaclonal-targeted mutagenesis or the invasion of the cells in the adjacent layer. However, invasion from neighbouring cell layers to make a sector in a flower has been described for chimaeric mutants by transposons^30^, and we did not observe any somaclonal-targeted mutagenesis or secondary mutagenesis, suggesting that sectors in a flower of plants #8-1 and #36-2 are caused by the invasion.

The nucleotide sequencing of the mutated region revealed variable mutations in the *InDFR-B* region, ranging from one bp insertion to deletions of up to 98 bp (Fig. 1e and Supplemental Fig. S2). In general, random NHEJ occasionally generates mutations without losing gene function, such as in-frame mutations (multiple of −3 nt deletion) or silent mutations (without changes in the amino acid sequence). In the present study, bi-allelic mutations of 3-bp deletions resulted in the leucine 197 deletion, observed in two independent plants (#9-1 and #39-1). The fact that neither mutant could produce anthocyanin suggested that leucine 197 is indispensable for the enzymatic activity of InDFR-B. In the grape DFR^48^, crystallization analysis revealed that the conserved proline 190 (corresponding to proline 195 in InDFR-B, next to the deleted leucine 197) is used for the interaction of flavonol with nicotinamide adenine dinucleotide phosphate (NADP^+^) to form the catalytic complex (Supplemental Fig. S4)^33^. The deletion of this amino acid leucine 197 might result in the failure of forming stacked saturated and aromatic rings, with lost enzyme activity. In this case, we successfully increased the efficiency of the targeted mutagenesis by selecting the CRISPR/Cas9 target sequence at the active site encoding the sequence of the target enzyme.

In the present study, we successfully applied a CRISPR/Cas9 system in *I. nil* for targeted mutagenesis in one generation. To our knowledge, this report is the first to examine changing flower colour using the CRISPR/Cas9 system. The successful results of the present study will facilitate the modification of flower colours and shapes with targeted mutagenesis in *I. nil* and other ornamental flowers or vegetables. Moreover, in the present study, we obtained T2 plants with targeted mutagenesis at the *InDFR-B* locus but without T-DNA. These plants are biologically non-transgenic and could not be distinguished from other spontaneous mutants^38, 39^. We propose that these plants will attract the attention of the public and will improve understanding of NBT.

## Methods

### Plant materials and growth conditions

The seeds of *I. nil* cv. Violet (obtained from the NBRP “Morning glory”) were used throughout the experiments. The seedlings were grown on vermiculite fertilized with 1,000-fold diluted Hyponex 6–10–5 solution (Hyponex Japan, Tokyo, Japan) once a week under continuous light (60 mmol m^−2^ s^−1^) at 25 °C for two weeks. Those plants were transferred under short-day conditions (8 h light: 16 h dark) at 25 °C for two days for flower induction. The plants were then further cultivated under long-day conditions (14 h light: 10 h dark) at 25 °C. Immature fruits were collected two to three weeks after flower opening^7^. The immature embryos were isolated from sterilized immature fruits and cultured on an embryoid induction medium [MS medium with 3 mg L^−1^ α –naphthaleneacetic acid (NAA), 6% sucrose and 0.2% Gelrite® (Wako, Tokyo, Japan)] to form the somatic embryos.

### Vector construction and transformation

An all-in-one binary vector^49^ harbouring *sgRNA*, Friedrich Fauser’s *Cas9 (*FF*Cas9)* and *Neomycin phosphotransferase II (NPTII)* expression loci was constructed as previously described^50^. Briefly, two complementary oligo DNAs for the *DFR-B* target sequence (Forward: 5′ ATTGAAGCATCATACCACCACTAG 3′; Reverse: 5′ AAACCTAGTGGTGGTATGATGCTT 3′) were annealed at 95 °C for 5 min. The target sequence-cloning vector, pUC19_AtU6oligo, was digested with restriction enzyme *Bbs*I and ligated to the annealed oligo DNA. The cloning vector and binary vector, pZD_*AtU6gRNA*_FF*Cas9*_*NPTII*, were digested with the restriction enzyme I-*Sce*I, and the sgRNA expression cassettes of each vector were subsequently exchanged.

*Agrobacterium*-mediated transformation using an immature embryo-derived secondary embryo was performed as previously described^8^. *Rhizobium radiobacter* (*Agrobacterium tumefaciens*) strain LBA4404 harbouring a ternary plasmid for *virG* N54D^51^ was used for transformation. The *Rhizobium radiobacter* were grown overnight at 28 °C in an LB liquid medium containing antibiotics. The bacterial cells were collected by centrifugation, washed and suspended with the secondary embryoid formation (SEF) medium (MS medium with 0.5 mg L^−1^ NAA and 6% sucrose). Somatic embryos were soaked in the bacterial suspension for 5 min and transferred to plates of an SEF medium with 0.2% Gelrite® and 10 mg L^−1^ acetosyringone. After three days of co-cultivation, the somatic embryos were washed and transferred to an SEF selection medium containing 25 mg L^−1^ kanamycin and Augmentin (125 mg L^−1^ Potassium Clavulanate and 250 mg L^−1^ Amoxicillin; Glaxo SmithKline K.K., Uxbridge, UK). Two to three weeks after selection, they were transferred to an embryoid maturation and germination medium [MS medium with 0.2 mg L^−1^ indoleacetic acid, 2 mg L^−1^ benzylaminopurine, 3% sucrose and 1.2% agar] contained 50 mg L^−1^ kanamycin and Augmentin. After two to six months, when the shoots were regenerated, they were transferred to a hormone-free 1/2 MS medium with 25 mg^−1^ kanamycin and Augmentin. When the roots were induced on the regenerated shoots, plantlets were transplanted to moist vermiculite for acclimatization. As the transgenic plants directly germinated from kanamycin-resistant secondary embryos, we described these plants as the T1 generation. The validity of transformation was confirmed by PCR using total DNA extracted from young leaves and primers for *NPTII* (Forward: 5′ GAGGCTATTCGGCTATGACT 3′, Reverse: 5′ TCCCGCTCAGAAGAACTCGT 3′). Total DNA was extracted from the young leaves of plants using a previously described method^52^. PCRs were performed with GoTaq® Green Master Mix (Promega, Madison, WI, USA) on a thermal cycler, with initial denaturation at 95 °C for 2 min followed by 35 cycles at 95 °C for 30 s, 55 °C for 30 s and 72 °C for 1 min and a subsequent extension step at 72 °C for 5 min.

### CAPS analysis

DNA fragments of InDFRs were PCR-amplified using total DNA of transformants with GoTaq® Green Master Mix (Promega, Madison, WI, USA) and the specific primers (*InDFR-A* Forward: 5′ CATAAAACCATTAGACCTG 3′, *InDFR-A* Reverse: 5′ AAATAACATATTGAATTCTGC 3′; *InDFR-B* Forward: 5′ TGCGGTTACCAAGCTAACGAA 3′, *InDFR*-*B* Reverse: 5′ GTGATCATGTCCGCTAAACCA 3′; *InDFR-C* Forward: 5′ TTGCGGATTTCCCTATTGGAT 3′, *InDFR-C* Reverse: 5′ GTTCCCTATAGAGACCGGACA 3′, *phytoene synthase* [*InPSY*: AB499050] Forward: 5′ GTGCAGAGTATGCAAAGACG 3′, *InPSY* Reverse: 5′ GCCTAGCCTCCCATCTATCC 3′). The thermal cycles of the reaction were same as the *NPTII* reactions in *InDFR-B*, *InDFR-C* and *InPSY*. The PCR cycles for *InDFR-A* were as follows: initial denaturation at 95 °C for 2 min followed by 35 cycles at 95 °C for 30 s, 50 °C for 30 s and 72 °C for 1 min and a subsequent extension step at 72 °C for 5 min. The amplified DNA fragments were digested with *Spe*I and analysed via agarose gel electrophoresis.

### Sequencing analysis

The total DNAs and *InDFR-B* primers were the same as those used in the CAPS analysis. Primer sets for analyzing candidates of off-target mutation were listed in Supplemental Table S2. The PCRs were performed using Advantage® 2 Polymerase Mix (BD Biosciences Clontech, Palo Alto, CA, USA) on a thermal cycler, with an initial denaturation at 95 °C for 1 min followed by 35 cycles of 95 °C for 30 s and 68 °C for 1 min and a subsequent extension step at 68 °C for 1 min. The PCR products were cloned into the pGEM®-T Easy Vector (Promega, Madison, WI, USA) and were sequenced using a CEQ8000 automated DNA sequencer with a DTCS Quick Start Kit (Beckman Coulter, Fullerton, CA, USA). Nucleotide and amino acid sequences were analysed using GENETYX-MAC (Software Kaihatsu Co., Tokyo, Japan). The *I. nil* genome sequence was analysed using the NCBI BLAST system 2.2.26 (DNA Data Bank of Japan, Mishima, Shizuoka, Japan)^53^ to detect potential off-target sequences.

## Electronic supplementary material

Supplemental information
