# Supplementary material for: CRISPR/Cas9-mediated mutagenesis of the dihydroflavonol-4-reductase-B (DFR-B) locus in the Japanese morning glory Ipomoea (Pharbitis) nil
Source: Sci Rep. 2017 Aug 30;7:10028. doi: 10.1038/s41598-017-10715-1 (PMC5577235; doi:10.1038/s41598-017-10715-1)
Supplement: Supplementary file 1 — Supplemental information [file 41598_2017_10715_MOESM1_ESM.pdf]

Supplemental information

CRISPR/Cas9-mediated mutagenesis of the *dihydroflavonol-4-reductase-B (DFR-B)* locus in the Japanese morning glory *Ipomoea (Pharbitis) nil*

Kenta Watanabe[1], Anna Kobayashi[2], Masaki Endo[3], Kimiyo Sage-Ono[4], Seiichi Toki[3,5,6] & Michiyuki Ono[1,2,4*]

[1]Graduate School of Life and Environmental Sciences, University of Tsukuba, Tsukuba, Ibaraki 305-8572, Japan.
[2]College of Biological Sciences, School of Life and Environmental Sciences, University of Tsukuba, Tsukuba, Ibaraki 305-8572, Japan.
[3]Plant Genome Engineering Research Unit, Institute of Agrobiological Sciences, National Agriculture and Food Research Organization, 2-1-2 Kannondai, Tsukuba, Ibaraki 305-8602, Japan.
[4]Gene Research Center, Tsukuba Plant Innovation Research Center (T-PIRC), University of Tsukuba, Tsukuba, Ibaraki 305-8572, Japan.
[5]Graduate School of Nanobioscience, Yokohama City University, 22-2 Seto, Kanazawa-ku, Yokohama, Kanagawa 236-0027, Japan.
[6]Kihara Institute for Biological Research, Yokohama City University, 641-12 Maioka-cho, Yokohama, Kanagawa 244-0813, Japan.

*Corresponding author: Michiyuki Ono
Phone number: +81-29-853-7759
E-mail address: ono.michiyuki.fm@u.tsukuba.ac.jp

Table S1. Potential off-target sites of *InDFR-B* knock out in the present study. The off-target sequences except *DFR*s were searched with GGGenome (http://gggenome.dbcls.jp/) against *I. nil* cv. TKS whole genome. Blue letters indicate PAM sequence. Red lower case letters show mismatches and gaps to the 23 bp on-target (*DFR-B*) sequence. Green letters indicate the 12 bp seed sequences. Search conditions used were 12 bp seed: 12 bp seed sequence allowing no mismatch, and <3 bp Mismatch: allowing less than 3 bp mismatches within 23 bp. Only sequences having a PAM sequence were selected. Candidate sites were numbered 1 to 9 for further analysis.

	candidate number	scaffold	strand	potision	sequence	PAM	mismatch(es) initial 8 bp	mismatch(es) total
DFR-B		805	+	1966837	AAGCATCATACCACCACTAGTGG	o	0	0
DFR-A		805	+	1959514	cAGCATCATACCACCACTAGTca	x		
DFR-C		805	+	1971365	cAGtATCATACCACCACTAGTca	x		
12 bp seed	1	875	+	640822	tgcacctcTACCACCACTAGTGG	o	0	8
	2	2036	+	2396563	ggGCccCgTACCACCACTAGGGG	o	0	5
<3 bp Mismatch	3	875	+	1857780	AAGCATaATACCACCACTAGTGG	o	1	3
					t g			
	4	2690	+	1189128	AA-tATCATACCACCAtTAGAGG	o	1	2
	5	2704	+	1868233	AAtCATCATACCACCAC-AGTGG	o	1	3
					t			
	6	3195	+	433510	AAGCcTCtTACCA-CACTAGCGG	o	1	3
	7	465	-	1708179	AAGCATaATACCACCACTAGTGG	o	1	3
					t g			
	8	1026	-	3378314	AA-CA-CATACCACCtCTAGCGG	o	1	3
	9	3054	-	232458	AAt-ATCATACCACCAtTAGAGG	o	1	2

Table S2. Primer sets used for DNA sequencing analysis of candidate off-target sites listed in Table S1. Primers were used for PCR amplification of the sites containing genomic DNA fragment as well as primers for DNA sequencing. *A nested PCR was used for specific amplification of the candidate 1 containing genomic DNA fragment. For the candidate 1, only 2nd PCR primers were used for DNA sequencing.

number of off-target candidate	direction	sequence
candidate 1 (for 1st PCR)*	Fw	5' ATATATTTTCTGTTGTGACC 3'
	Rv	5' TCTTCTCCTGGCTTTTGA 3'
candidate 1 (for 2nd PCR)*	Fw	5' AATATTTATCTTAATTGCAG 3'
	Rv	5' TACCAAATCAAAATGCTC 3'
candidate 2	Fw	5' CCTTAGGGAATGAATTGAGC 3'
	Rv	5' TAGGCAGAGTAATCAGTCCA 3'
candidate 3	Fw	5' ATAAGTGCTTGTTTCACCA 3'
	Rv	5' GTAATGTTATTTTGGACCCT 3'
candidate 4	Fw	5' GCCTATTCCGTGAACCAA 3'
	Rv	5' GTTGTGAAACTGCGTCCA 3'
candidate 5	Fw	5' TGATTTGCGAGAACAAGCTG 3'
	Rv	5' GTACAACTTTAGAAGCGTGGA 3'
candidate 6	Fw	5' TCAGCCGGGTTCAAATCGTC 3'
	Rv	5' TACCGTAGTACTCGCTTCACC 3'
candidate 7	Fw	5' ATAACTGCTTGTTTCACCA 3'
	Rv	5' TTTTCATGTTCAAAGGTGAC 3'
candidate 8	Fw	5' CATATATGCTCTAGAAAGACG 3'
	Rv	5' TGAAACCCTTTGAGGTGA 3'
candidate 9	Fw	5' GAAGCAGTAAGCTATTCCCA 3'
	Rv	5' ATAATTTGTATATTGCCGGAT 3'

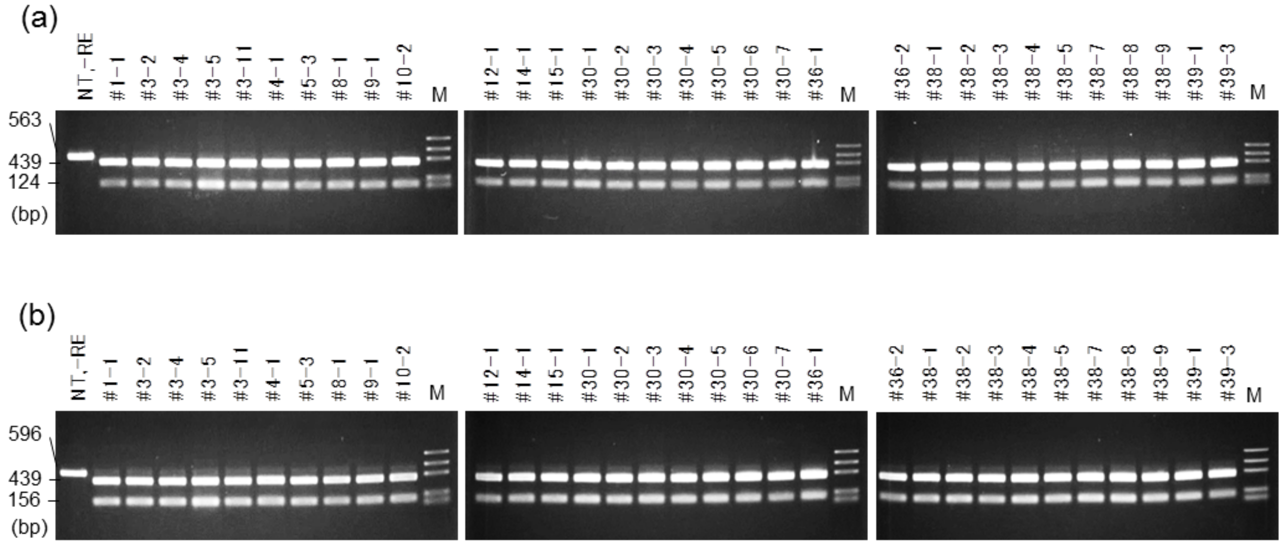

Figure S1. CAPS analysis of the *InDFR-A* and *InDFR-C* loci in transformed plants using the CRISPR/Cas9 system. Total DNA was extracted from the leaves of the transformed plants and amplified through PCR. The PCR products were digested using the *Spe*I restriction enzyme, except NT, -RE. (a) All of the PCR fragments of *InDFR-A* 563 bp in length were cleaved into 439- and 124-bp fragments using *Spe*I. (b) All of the PCR fragments of *InDFR-C* 596 bp in length were cleaved into 439- and 156-bp fragments. The results indicated that there were no off-target mutations in *InDFR-A* and *InDFR-C* using sgRNA of *InDFR-B*. M: marker (1,000, 700, 500, 200 and 100 bp); NT, -RE: PCR product of NT without restriction enzyme digestion.

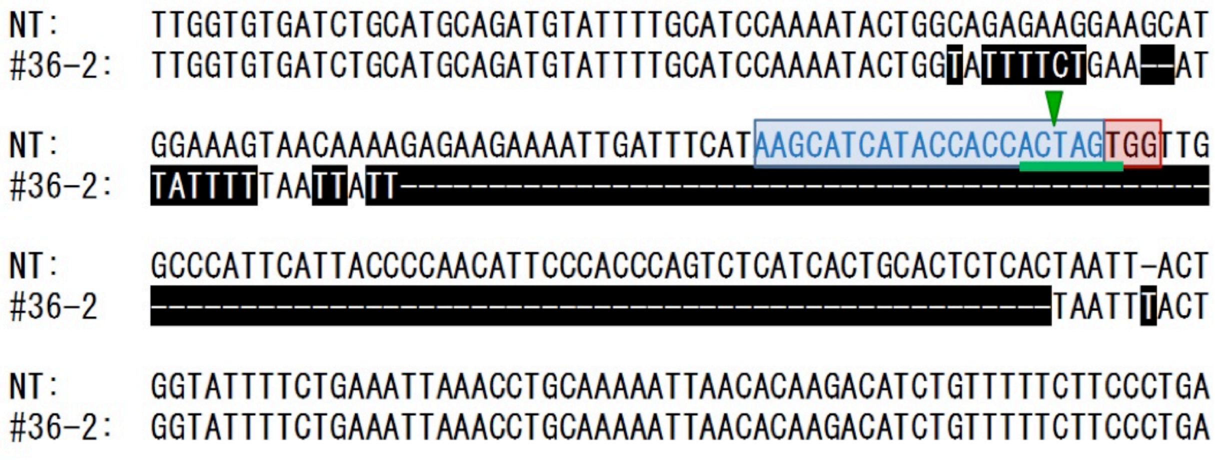

Figure S2. DNA sequence of *InDFR-B* with a long deletion identified from plant #36-2. The NT type sequence is shown above. The red box indicates the PAM sequence, the blue box indicates the target sequence and the *Spe*I restriction enzyme site (ACTAGT) is shown with green underline. The green triangle indicates the expected cleavage site using the CRISPR/Cas9 system. Deletions, insertions and point mutations are shown with black highlight.

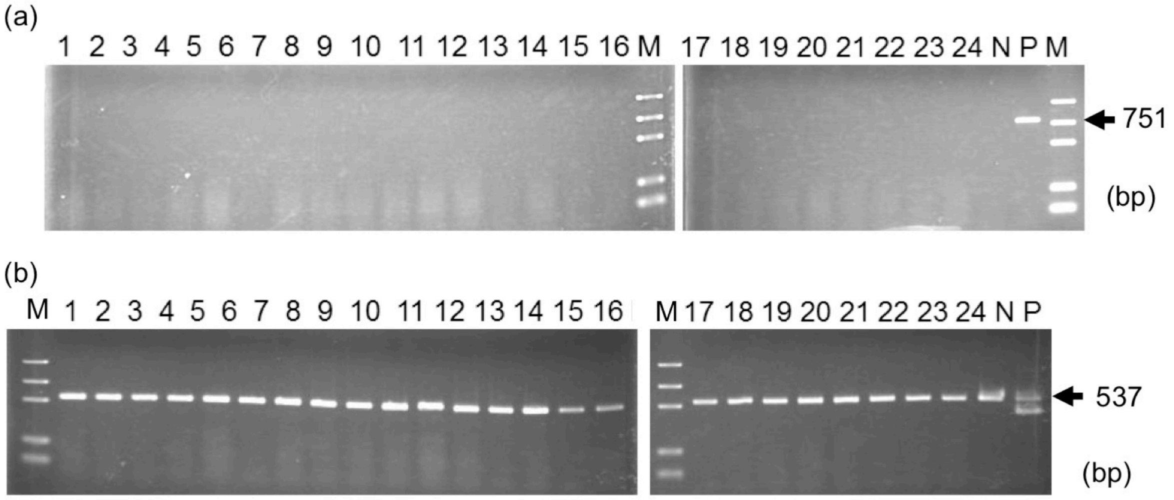

Figure S3. Genotype evaluations of the T2 progeny of chimaeric plant #36-2. (a) The *NPTII* gene was PCR-amplified and analysed via electrophoresis. (b) An internal control for the PCR using the *DFR-B* gene. N: non-transgenic *I. nil* cv. Violet. P: parental transgenic plant line #36-2 (T1 generation). Arrows indicate the positions of amplified fragments of *NPTII* (751 bp) and *DFR-B* (531 bp), respectively. The lower band of P in (b) is the deleted fragment of *DFR-B* (433 bp). M: marker (1,000, 700, 500, 200 and 100 bp).

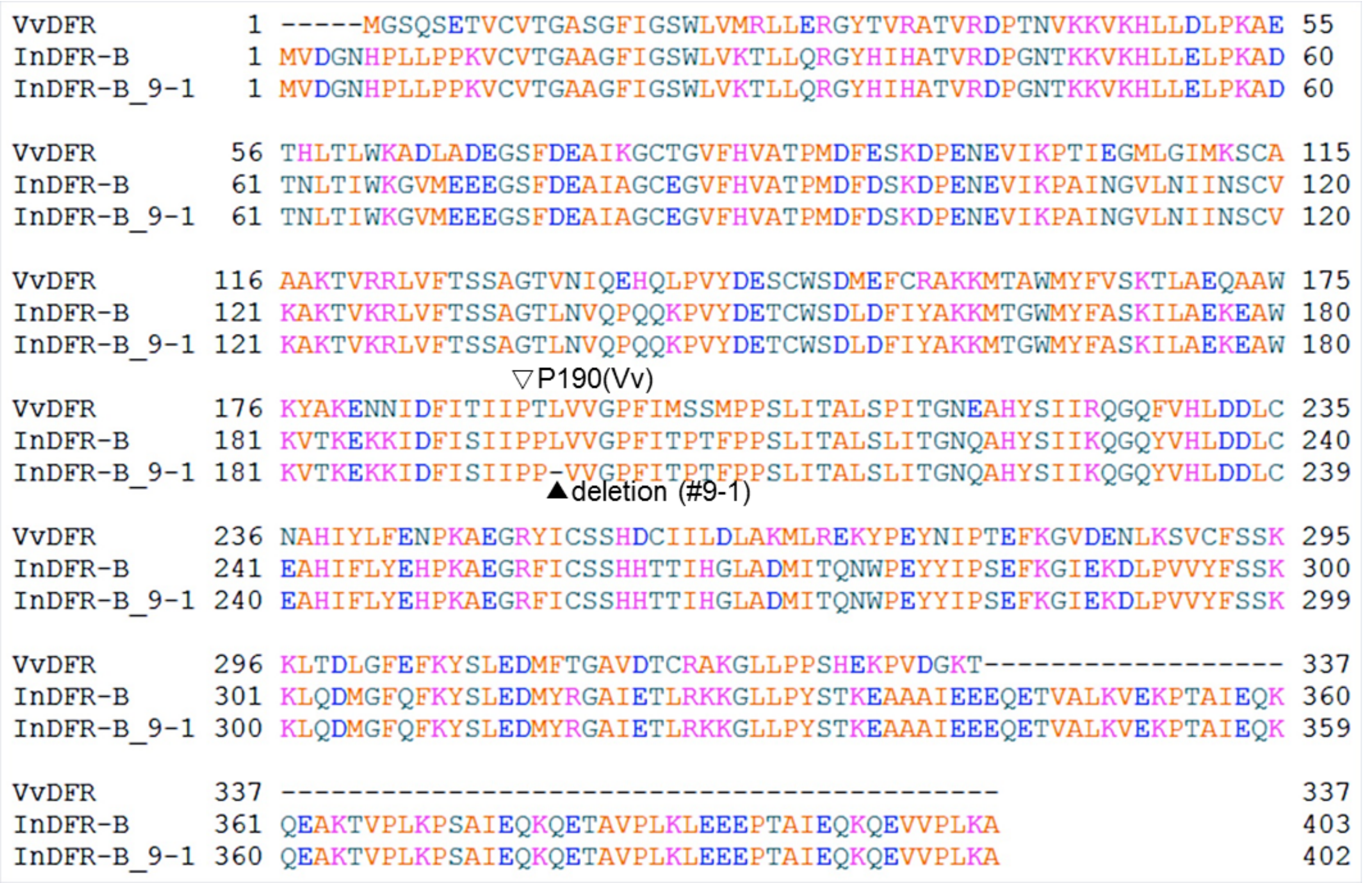

Figure S4. Deletion of a single amino acid (L197) abolished enzymatic activity. A multiple sequence alignment of the DFR amino acid sequence. Amino acid residues are presented with single letters coloured based on similarity. VvDFR: DFR protein of grape *Vitis vinifera* (GenBank accession X75964)[1]. InDFR-B: DFR-B wild-type protein of *I. nil* (DDBJ accession AB006793)[2]. InDFR-B_9-1: A mutated DFR-B protein of plant #9-1 in this study. The black triangle shows the deletion of leucine 197 (L197). The open triangle shows the conserved proline 190 (P190) of VvDFR that essential for forming the DFR–NADP+–flavonol complex[3].

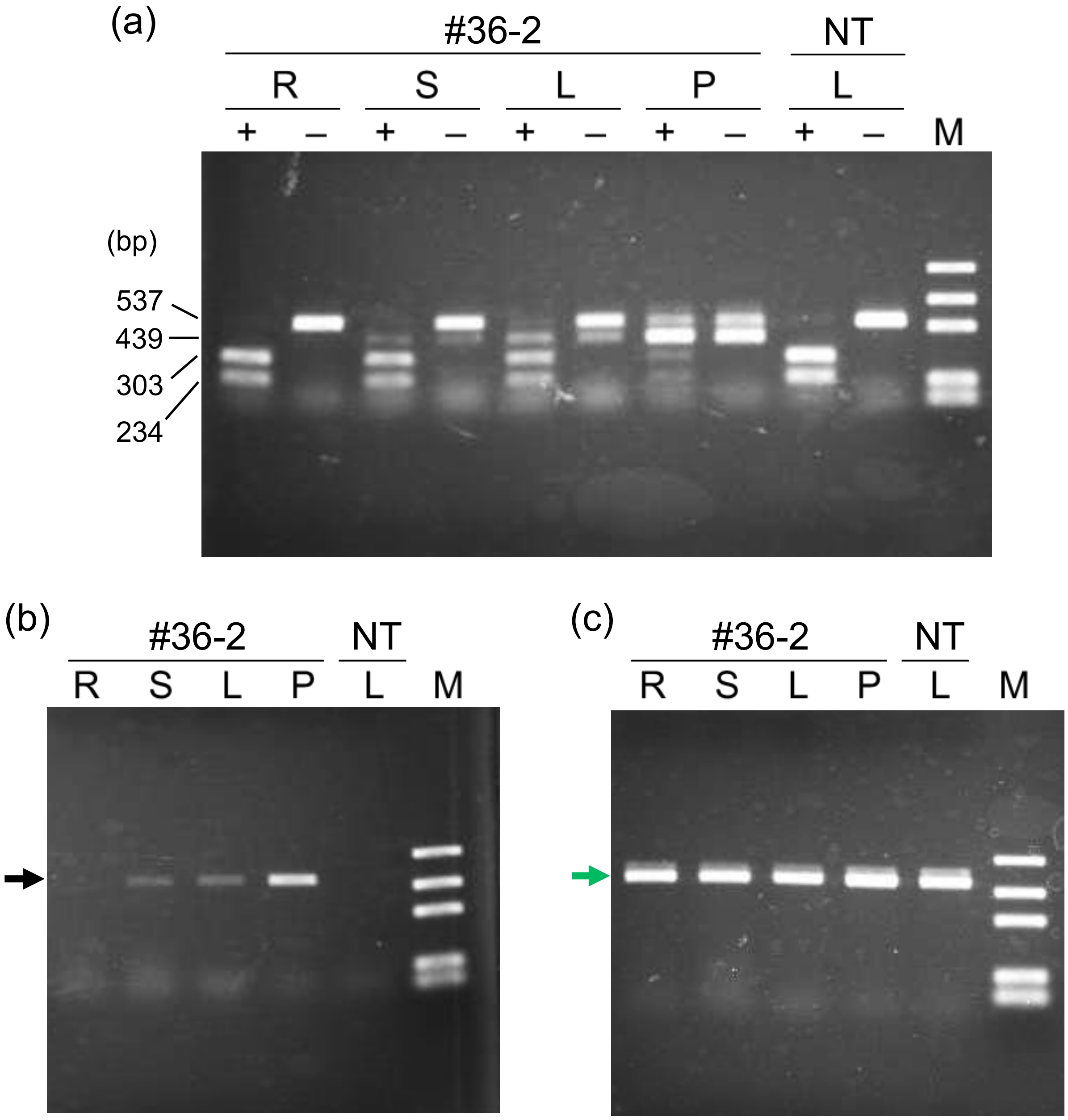

Figure S5. Organ-specific CAPS analysis of the *InDFR-B* and genotype analysis in periclinal chimaeric plant #36-2. Total DNAs were extracted from the root (R), stem (S), leaf (L) and petal (P) of plant #36-2 and leaves of non-transformant (NT) plant, and amplified by PCR reaction. (a) The PCR fragments of *InDFR-B*. The PCR products were then digested using the *Spe*I restriction enzyme (+). -: without *Spe*I restriction enzyme digestion. (b, c) The PCR fragments of *NPTII* (black allow; a transgene) and *phytoene synthase* (green arrow; an internal control) respectively. M: marker (1,000, 700, 500, 200 and 100 bp) .

The root of #36-2 did not show DNA bands corresponding to the mutated *dfr-b* [a, 537 (+) and 439 bp] and the *NPTII* (b, black arrow) fragments. Moreover, the stem, leaf and petal showed these bands, however, these bands of the stem and leaf were weaker than these bands of petal, while the bands of the internal control gene (c, green arrow) were similar levels. The band depth of mutated *dfr-b* and *NPTII* reflected the ratio of L1 derived tissues among total tissues used for DNA extraction. A root consists of L3 layer only, while upper ground tissues contain L1, L2 and L3 tissues but with different ratios. Stem and leaf consist of mainly pith (L3 derived) and petal has thick L1 epidermis. These results strongly support that plant #36-2 is an L1 periclinal chimeric mutant.

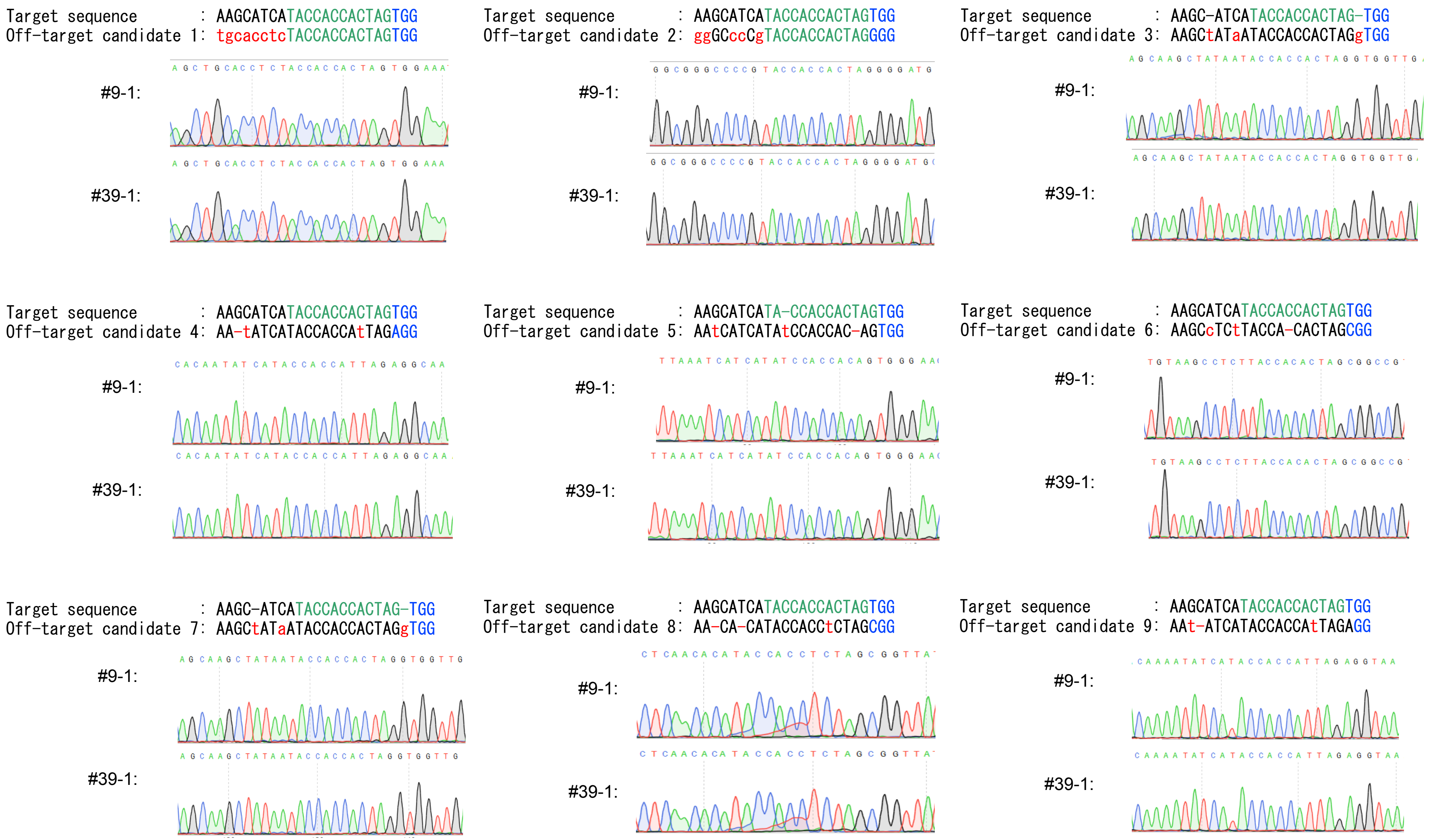

Figure S6. Sequence analysis of the off-target mutation candidates 1 to 9 in the plant #9-1 and #39-1. There was no altered sequence. We concluded that these sites are no more candidates of off-target mutation at least in these plants.

References

1. Sparvoli, F., Martin, C., Scienza, A., Gavazzi, G. & Tonelli, C. Cloning and molecular analysis of structural genes involved in flavonoid and stilbene biosynthesis in grape (*Vitis vinifera* L.). *Plant Mol. Biol.* **24**, 743-755 (1994).

2. Inagaki, Y. *et al*. Genomic organization of the genes encoding dihydroflavonol 4-reductase for flower pigmentation in the Japanese and common morning glories. *Gene* **226**, 181–188 (1999).

3. Trabelsi, N. *et al.* Structural evidence for the inhibition of grape dihydroflavonol 4-reductase by flavonols. *Acta Crystallogrphica* **D64**, 883–891 (2008).

4. Aida, R., Sasaki, K. & Ohtsubo, N. Production of chrysanthemum periclinal chimeras through shoot regeneration from leaf explants. *Plant Biotechnol*. **33**, 45–49 (2016).
